# Aminoquinolines as Translational Models for Drug Repurposing: Anticancer Adjuvant Properties and Toxicokinetic-Related Features

**DOI:** 10.1155/2021/3569349

**Published:** 2021-09-03

**Authors:** Paulo Michel Pinheiro Ferreira, José Roberto de Oliveira Ferreira, Rayran Walter Ramos de Sousa, Daniel Pereira Bezerra, Gardenia Carmen Gadelha Militão

**Affiliations:** ^1^Department of Biophysics and Physiology, Laboratory of Experimental Cancerology (LabCancer), Federal University of Piauí, Teresina 64049-550, Brazil; ^2^Center for Integrative Sciences, State University of Health Sciences of Alagoas, Maceió 57010-382, Brazil; ^3^Gonçalo Moniz Institute, Oswaldo Cruz Foundation (IGM-FIOCRUZ-BA), Salvador 40296-710, Brazil; ^4^Department of Physiology and Pharmacology, Federal University of Pernambuco, Recife 50670-901, Brazil

## Abstract

The indiscriminate consumption of antimalarials against coronavirus disease-2019 emphasizes the longstanding clinical weapons of medicines. In this work, we conducted a review on the antitumor mechanisms of aminoquinolines, focusing on the responses and differences of tumor histological tissues and toxicity related to pharmacokinetics. This well-defined analysis shows similar mechanistic forms triggered by aminoquinolines in different histological tumor tissues and under coexposure conditions, although different pharmacological potencies also occur. These molecules are lysosomotropic amines that increase the antiproliferative action of chemotherapeutic agents, mainly by cell cycle arrest, histone acetylation, physiological changes in tyrosine kinase metabolism, inhibition of PI3K/Akt/mTOR pathways, cyclin D1, E2F1, angiogenesis, ribosome biogenesis, triggering of ATM-ATR/p53/p21 signaling, apoptosis, and presentation of tumor peptides. Their chemo/radiotherapy sensitization effects may be an adjuvant option against solid tumors, since 4-aminoquinolines induce lysosomal-mediated programmed cytotoxicity of cancer cells and accumulation of key markers, predominantly, LAMP1, p62/SQSTM1, LC3 members, GAPDH, beclin-1/Atg6, *α*-synuclein, and granules of lipofuscin. Adverse effects are dose-dependent, though most common with chloroquine, hydroxychloroquine, amodiaquine, and other aminoquinolines are gastrointestinal changes, blurred vision ventricular arrhythmias, cardiac arrest, QTc prolongation, severe hypoglycemia with loss of consciousness, and retinopathy, and they are more common with chloroquine than with hydroxychloroquine and amodiaquine due to pharmacokinetic features. Additionally, psychological/neurological effects were also detected during acute or chronic use, but aminoquinolines do not cross the placenta easily and low quantity is found in breast milk despite their long mean residence times, which depends on the coexistence of hepatic diseases (cancer-related or not), first pass metabolism, and comedications. The low cost and availability on the world market have converted aminoquinolines into “star drugs” for pharmaceutical repurposing, but a continuous pharmacovigilance is necessary because these antimalarials have multiple modes of action/unwanted targets, relatively narrow therapeutic windows, recurrent adverse effects, and related poisoning self-treatment. Therefore, their use must obey strict rules, ethical and medical prescriptions, and clinical and laboratory monitoring.

## 1. Introduction

Globally, about 1 in 6 deaths is due to cancer and about 70% of cancer deaths occur in low- and middle-income countries. Approximately, one third of these deaths are associated with high body mass index, low consumption of fruits and vegetables, lack of physical activity, and use of tobacco and alcohol. Tobacco use is the most important risk factor for cancer and accounts for approximately 22% of the total deaths. On the other hand, infections such as hepatitis and human papilloma virus (HPV) are responsible for up to 25% of cancer cases in poor and developing countries. In 2018 alone, approximately 9.6 million deaths were related to cancer [1–3].

Determining treatment and palliative care goals are critical steps for cancer therapy with integrated and people-centered health services [3]. Even with a variety of options to treat sarcomas, carcinomas, and adenocarcinomas, such as antimetabolites, microtubule inhibitors, DNA intercalators [4], and monoclonal antibodies [5], resistance remains the cause central to therapeutic failures as well as adverse side effects [6].

In this context, the synthesis and identification of strategic molecules is essential if we want low cost, efficiency, and speed in the production of valuable chemotherapy molecules. Here, we can include aminoquinoline compounds that have the amino group at position 4 of the quinoline ring system. These compounds include molecules used in the treatment of first line (amodiaquine and chloroquine), recurrence (tafenoquine), uncomplicated (hydroxychloroquine) and prevention (chloroquine, hydroxychloroquine and tafenoquine) of malaria infections by *Plasmodium vivax*, *P. malariae*, *P. ovale*, and *P. falciparum* [7–9].

In December 2019, a new severe acute respiratory syndrome coronavirus-2 (called SARS-CoV-2) emerged in China and led to the coronavirus-related pandemic in 2019 (COVID-19) [10]. The indiscriminate use of chloroquine and hydroxychloroquine as a first-line, adjuvant, or palliative drug(s) to treat victims of COVID-19 or to control new local outbreaks as a prophylactic [11, 12] emphasized the various longstanding clinical branches of drugs, including those against chronic disorders. Advantages such as low cost, long usage history, and market availability even in developing countries where malaria is endemic are reasons that explain, at least in part, the commercial triumph of these drugs, converting 4-aminoquinolines into “star drugs” for their reuse in the pharmaceutical industry. Then, we performed a review on the anti-tumor mechanisms of aminoquinolines, focusing on the responses and differences of histological tumor tissues and on the aspects of toxicity related to pharmacokinetics.

To carry out a comprehensive and consistent analysis, we use only primary and secondary materials, including research articles, reviews, books, and government publications written in English, Portuguese, or Spanish. The bibliographic research was performed in the scientific databases ScienceDirect, Scopus, PubMed, and Scielo. The descriptors “autophagy,” “cell cycle,” “apoptosis,” “drug repurposing”, and “anti-tumor” were combined with “aminoquinoline” for a narrative scientific exploration.

## 2. Main Text

### 2.1. Drug Repurposing for Anticancer Agents: Need or Pharmaceutical Business?

Less toxic and more effective treatment designs are often the main reasons for redirections, considering previously recorded aspects of preclinical and clinical pharmacodynamics and toxicokinetics, making drug reuse faster [13–15].

Examples of reuse of effective anticancer drugs in advanced preclinical or clinical studies are almost immeasurable. As a typical example, thalidomide is a leading molecule that has been marketed in 1956 in West Germany, first as antiflu and in 1957 as an antiemetic for pregnancy, but has now been repurposed and approved for multiple myeloma [16, 17]; itraconazole, a triazole antifungal developed in the 1980s, showed anticancer activities in preclinical *in vitro* and *in vivo* models of pancreatic ductal adenocarcinoma derived from liver metastasis [18]; disulfiram, initially approved to mitigate alcoholism, has been investigated to treat radiation-resistant breast cancer stem cells [19]; nelfinavir, originally indicated for the treatment of HIV infection, also exhibits synergistic effects against human cervical cancer cells [20]; sildenafil, which failed in phase II clinical trials for angina disorders, has been redirected to the treatment of erectile dysfunctions and sensitizes prostate cancer cells to doxorubicin-mediated apoptosis [21]; mebendazole, a broad spectrum anthelmintic developed for the treatment of veterinary parasites, has advanced from the treatment of animals to the first clinical applications in humans, inhibiting the growth of adrenocortical carcinoma, gastric cancer, medulloblastoma, glioblastoma, leukemia and myeloma, and breast and prostate cancers [22]; metformin, a classic hypoglycemic medication for diabetes, has revealed a new identity as an antitumor activity by suppressing the mammalian target of rapamycin (mTOR) in human cervical cancer [20] and acute myeloid leukemia [23], and valproic acid, an anticonvulsant that has been considered in several clinical trials due to its epigenetic properties, inhibition of histone deacetylase, and induction of autophagy in neoplastic stomach cells [24].

**Figure 1:**
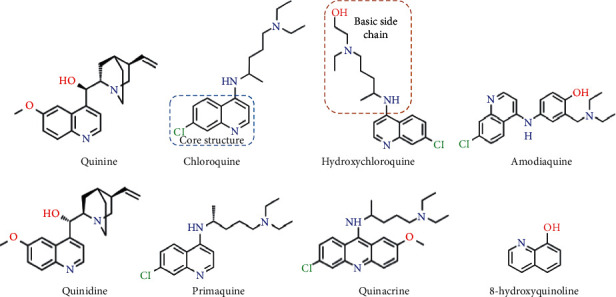
Structures of some common aminoquinolines in clinical use as antimalarials or anti-inflammatory drugs and under investigations as anticancer agents.

In 1934, the first synthetized aminoquinoline—chloroquine [4-N-(7-chloroquinolin-4-yl)-1-N,1-N-diethylpentane-1,4-diamine]—was based on the quinine structure isolated from Cinchona officinalis barks in the 1800s. In 1946, hydroxychloroquine [2-[[(4S)-4-[(7-chloroquinolin-4-yl)amino]pentyl]-ethylamino]ethanol] was synthesized, and both molecules were developed as antimalarial tools (Figure 1), as well as extra 4- and 8-aminoquinolines (amodiaquine, tafenoquine, primaquine, mefloquine, quinacrine, quinine, quinidine, and 8-hydroxyquinoline and artemisinin) in an attempt to overcome resistance in *Plasmodium* species, and the side effects [8, 25, 26] seem similar to that of cancer therapy, whose initial successful single-target therapies have been replaced by more combined efficient protocols.

Currently, the aminoquinolines, chloroquine phosphate and hydroxychloroquine sulphate, have been the most common salts used [27] as the first-line regimen for the radical cure of malaria by *P. vivax* in most regions [26] and to treat acute and chronic inflammatory conditions [9, 28–30], respectively, although primaquine and amodiaquine, when used alone or in combination with artemisinin, provide adequate efficacy against many chloroquine-resistant parasites [8, 26].

### 2.2. Antiproliferative Mechanism of Aminoquinolines

More than 50 years ago, chloroquine showed promising cytotoxicity of tumor cells *in vitro* [31], but only in the last two decades, studies with chloroquine, hydroxychloroquine, and related molecules demonstrated lysosomal-mediated cell death in cancer cells. The exact mechanism of cytotoxic action is not yet fully understood, but hypothesis have attempted.

These molecules can enter into endosomes/lysosomes by passive diffusion, or they can be taken up together with sodium in the exchange of protons, as demonstrated by specific inhibitors of eukaryotic membrane Na^+^/H^+^ exchangers (NHE) [32]. Both converge in the accumulation within endosomes/lysosomes, leading to the interference of the autophagic flux [33–35], disruption of several enzymes (e.g., acid hydrolases and cathepsin B and D lysosomal cysteine proteases) [36, 37], inhibition of antigen processing [38, 39], and post-translational modification of recently produced proteins [35, 40]. In addition, preclinical and clinical investigations are testing the effectiveness of quinines as inhibitors of the autophagy flux to overcome resistance when traditional chemotherapy drugs are used as monotherapy, since the induction of autophagy has been associated with resistance in the therapy of cancer [41, 42].

#### 2.2.1. Brain Tumor Cells

**Figure 2:**
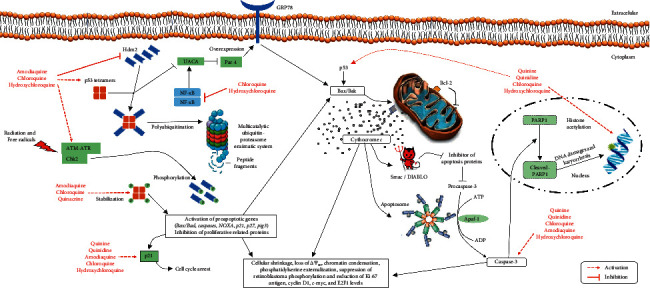
Pathways involved in general cytotoxicity and activation of apoptosis by 4-aminoquinolines. The transportation of both drugs is completely via passive diffusion (i.e., no transporters are involved). Bak and Bax are p53-induced proapoptotic members that constitute the apoptotic pore complex for the release of mitochondrial cytochrome c, leading to mitochondrial depolarization, activation of caspase-3, cleaving of poly-(ADP-ribose)-polymerase 1 (PARP-1), and nuclear DNA fragmentation. Aminoquinoline-dependent DNA damage activates p53 and its downstream gene p21, resulting in cell cycle arrest after a post-translational p53 activation by phosphorylation of the ataxia telangiectasia-mutated (ATM) protein, leading to ATM-dependent phosphorylation of p53 checkpoint protein kinase. Moreover, chloroquine, quinacrine, and amodiaquine trigger p53 stabilization in TP53-specific reporter human cancer cells and block the p53 ubiquitination properties of human double minute 2 (Hdm2) molecules, which in turn prevents p53 proteasome degradation. Stimulation of histone acetyltransferase (HAC) and inhibition of histone deacetylase (HDAC) are part of the rationale pattern of arresting growth. Chloroquine is linked to the activation of p53, inhibition of NF-*κ*B (factor nuclear kappa B) and uveal autoantigen with coiled-coil domains and ankyrin repeats (UACA), which promotes secretion of prostate apoptosis response-4 (Par-4) and expression of glucose regulated protein 78 (GRP78) receptor on the cancer cell surface, and consequent apoptosis.

Chloroquine decreases cell proliferation of p53 wild-type glioma lines more efficiently, indicating a key p53 responsibility for apoptotic cell death and cell cycle control through the HDM2, P21, PIG3, and *BAX* genes (Figure 2). Likewise, the induction of apoptosis *in vivo* was found in mice with U87MG glioma intracranially when treated with chloroquine [43].

On the other hand, chloroquine-induced neuronal cell death of normal neurons [44] indicates mitochondrial dysfunction as a result of p53-independent effects [43], but dependent on cathepsin D lysosomal cysteine proteases processing, proposing direct or indirect actions on the cathepsin D metabolism [37]. Moreover, chloroquine activation of ataxia telangiectasia-mutated (ATM)/ataxia telangiectasia and Rad3-related (ATR) kinase DNA injuries [45] seems unnecessary for caspases and p53 activation [37, 43, 44, 46–48], suggesting that aminoquinolines induce the death of glioblastoma cells, regardless of the p53 status [37].

The absence of DNA damage induced by chloroquine similar to DNA damage by direct ionizing radiation with consequent activation of p53 can be associated with its mechanism of interaction with DNA molecules, since chloroquine intercalates into DNA, but does not cause DNA damage directly [43, 49]; this does not exclude that caspase-3 activation is stronger in wild-type p53 glioma cells, proposing a clear contribution of p53 to chloroquine-induced apoptosis [43].

U251-MG brain cell line, orthotopic GL-261 gliomas, or rat brain-implanted C6 cells treated with suberoylanilide hydroxamic (histone deacetylase inhibitor, HDACi) and temozolomide (alkylating agent) in the presence of chloroquine 10–15 *μ*M showed reduced cell viability, morphology changes, increase in the sub-*G*_1_ population, Bax, cleaved-caspase-3, and cleaved-PARP1 [poly-(ADP-ribose)-polymerase 1], externalization of phosphatidylserine, and activation of caspase-3/7 (Table 1). Such events are features of apoptosis, but the time course curves showed that the *G*_2_/M arrest occurs with autophagy and before the apoptosis because the blocking of this response with autophagy inhibitors (3-methyladenine and chloroquine, for example) makes cells susceptible to temozolomide and suberoylanilide hydroxamic [54, 60].

#### 2.2.2. Human Cervical Tumors

**Figure 3:**
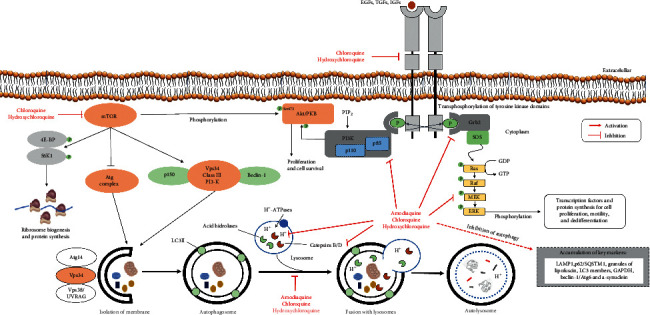
Molecular findings, which support the theory that chloroquine and analogues exerts, at least in part, antineoplastic effects altering the phosphorylation *status* of EGFR/PI3K/Akt/mTOR/Atg and p53 pathways, inhibiting directly PI3K-Akt and mTOR kinases, obstructing catalytic subunits in the ATP-binding site or altering the recycling of tyrosine kinase receptors, besides impairing or interfering in lysosomal and autophagosome functions. mTOR phosphorylates the eukaryotic initiation factor 4E-binding protein (4E-BP) and the p70S6 kinase1 (S6K1). Therefore, if specific drug inhibitors against mTOR kinase are used, this should not only have altered proliferation but also the protein synthesis rate. Vesicular protein sorting 34 (Vps34) complex I has Atg14p as an additional factor, which participates in the formation of autophagosomes, while complex II has Vps38, which is required for vacuolar protein sorting. These catalytic complexes work as ubiquitin-like conjugation systems for phagophore elongation and recruitment of other proteins to the self-digesting process, as seen with Vps34, a phosphatidylinositol serine-threonine kinase, its binding partner Beclin-1 (Atg6) and the protein kinase p150 in mammals (Vps15). Assembly of this complex is crucial for autophagy and it recruits other proteins to the phagophore assembly site (PAS). Therefore, the phagophore elongates into a cup-shaped structure and begins to engulf cellular material, sequestering the material in a double-membraned autophagosome. Both chloroquine and hydroxychloroquine block autophagy in initial phases, causing accumulation of acidic vesicle cell markers and appear to deactivate upstream members of mitogen-activated protein kinase (MAPK) pathway, preventing phosphorylation and activation of extracellular signal-regulated kinases (ERK)1/2 by a paradoxical phosphorylation of Raf at specific residues, which possibly blocks ERK activation by Akt activity.

Human papilloma positive HeLa cells (p53 wild-type) are resistant to apoptosis-inducing effects of death receptors [64], but pretreatment with 75 *μ*M chloroquine sensitized HeLa cells towards apoptosis mediated by Fas, as measured by TUNEL staining of DNA strand breaks [65], due to the disruption of mitogen-activated protein kinases (MAPK)/extracellular signal-regulated kinases (ERK)1/2, as found in cells treated with PD98059, a MEK1 inhibitor. Indeed, chloroquine and analogues appear to disable members upstream of the MAPK pathway (Figure 3), avoiding ERK phosphorylation and activation by a paradoxical Raf phosphorylation in specific residues, which possibly blocks the ERK activation by Akt activity [65].

HELa cells treated with 10–30 *μ*g/mL of hydroxychloroquine presented an increase in lysosomal volume and cathepsin B release from lysosomes to the cytosol and the nucleus, resulting in cytoplasmic vacuolization, cellular shrinkage, exposure of phosphatidylserine, loss of mitochondrial transmembrane potential (ΔΨ*m*), release of cytochrome c, activation of caspase-3 (Figure 2), and condensation of chromatin. In particular, vacuolization was found before chromatin condensation and was accompanied by the signs of macroautophagy [36]. These effects were blocked by bafilomycin A1, which prevents degradation of LC3, induces its accumulation in autophagolysosomes [66] and acts as an inhibitor of the vacuolar-type H^+^-ATPase, changing endosomal pH [67], showing that hydroxychloroquine activated apoptosis via lysosomes instead of other organelles (mitochondria or nuclei, for example).

The colorimetric MTT assay indicated that 3-methyladenine (3-MA) or chloroquine separately has no significant effects on the viability of HeLa cells, but both enhance the cytotoxic effects of cisplatin. The cotreatment also increased the expression of p62, the levels of cleaved caspase-3/-4, caused inhibition of autophagy downstream, and accumulation of ubiquitinated beclin-1 and LC3II misfolded proteins, and almost simultaneous apoptotic activation. Since cisplatin induces the generation of misfolded proteins, but increases autophagy, this would alleviate the physiological stress of endoplasmic reticulum by clearing the ubiquitinated proteins, which would trigger intrinsic apoptosis in HeLa cells [55]. The compound 3-MA is an inhibitor of phosphatidylinositol 3-kinases, which play an important role in controlling the activation of mTOR, a key regulator of autophagy [68].

#### 2.2.3. Colorectal Cancers

As a pyrimidine analogue, 5-fluorouracil (5-FU) acts as an antimetabolite to inhibit DNA and RNA synthesis, but it also has radiosensitizing, immunosuppressive, and mutational properties and has been widely used to treat various solid tumors, including colorectal, breast, stomach, pancreas, ovary, bladder, and liver cancers [69]. The apoptotic effects of 5-FU on human colorectal adenocarcinoma HT-29 cells were also increased by chloroquine. The pretreatment of HT-29 cells with chloroquine suppressed CDK-2 expression and catalytic activity of cyclin E/CDK2 complexes (Figure 2), leading to the (*G*_0_/*G*_1_) arrest [52]. Such findings suppose autophagy as a protective route against the action of 5-FU, since autophagic inhibitors increase the antiproliferative properties of this fluoropyrimidine.

Murine cell lines showing endogenous upregulation of receptor-interacting protein kinase 3 (RIP3) were more sensitive to chloroquine [61] and presented cytosolic accumulation of RIP3-p62 complexes and LC3-II, which is commonly recruited to phagosome membranes. However, initial/executioner caspase levels are apparently not altered by chloroquine during necroptotic cell death in CT-26 cells [61].

Since the morphological and flow cytometric investigations of chloroquine-treated CT-26 cells showed dissolved nuclei, condensation, swelling of organelles, and rupture of the cell membrane, these findings suggested that, instead of apoptosis, RIP3-dependent necroptosis was probably a reason for RIP3^+^-chloroquine-induced cell death [61, 70, 71].

The induction of tumor apoptosis *in vivo* exposed that apoptosis is not the only way by which chloroquine activates death cascade, as verified by TUNEL experiments and signals of necroptosis. In any case, mice with a CT-26-tumor xenograft showed tumor reduction after adjuvant treatments, whereas chloroquine alone showed a 45% reduction, and the combination with chemotherapies increased by up to 80% [61].

Chloroquine plus sunitinib, bevacizumab, and/or oxaliplatin increased intracellular levels of p62, indicating the accumulation and interruption of autophagic flux, increased caspase-3 activity and sensitivity under hypoxia conditions, and reduced blood vessel formation, expression of CD31, microvessel density, and nitric oxide levels in colorectal cancers [58, 59]. The growth of HT-29 colon cancer xenografts in bevacizumab- and oxaliplatin-treated mice was postponed from 7.2 to 23 days when bevacizumab and oxaliplatin were coadministered with chloroquine [58].

In addition to acting as inhibitors of autophagy, chloroquine, quinacrine, and amodiaquine trigger p53 stabilization in TP53-specific reporter human cancer cells [59] and wild-type cell lines [49, 72] (Figure 2). Amodiaquine *in vitro* at 20 *μ*M was specifically more efficient than chloroquine in inducing p53 stabilization by an independent ATM signaling pathway, interrupting cell proliferation of colorectal carcinoma cell lines (in addition to breast, hepatic, lung, sarcoma, and melanoma), decreasing the synthesis of a general ribosome precursor—47S rRNA—in U2OS cells, inducing the accumulation of LC3II autophagosome and lysosomal associated membrane protein 1 (LAMP1), and impairing translocation of the DDX21 nucleolar helicase to the nucleoplasma [49], the catalytic protein involved in the synthesis and processing of rRNA [73].

The nucleolar changes induced by amodiaquine were similar to those observed in cells treated with chloroquine and BMH-21l, a polymerase I inhibitor. Furthermore, amodiaquine inhibited the activity of ubiquitin ligase Hdm2's and thereby stabilized/activated p53 [49].

#### 2.2.4. Breast Carcinomas

Quinidine [74–76], quinine [77], chloroquine [76, 77], and hydroxychloroquine [75] induced differentiation in MCF-7 cancer cells, as demonstrated by the accumulation of cells in the *G*_0_ phase, intracellular milk fat globule membrane protein and lipid droplets (typical markers of differentiation), increased p21 and suppressed phosphorylation of retinoblastoma and expression of Ki-67 antigen, cyclin D1, *c*-myc, and E2F1 protein levels (Figure 2). While chloroquine was stronger in stimulating MCF-7 apoptosis, quinine was the most active in promoting differentiation [77].

Chloroquine or hydroxychloroquine + all-trans retinoic acid also reduced MCF-7 cells positive for Ki67, and their clonogenicity and hydroxychloroquine altered the acetylation *status* in the N-terminal lysines of the histones H3 and H4, epigenetic sites expected by the “zip”: model of histone acetylation [51]. These observations indicate that, in association with all-trans retinoic acid, quinidine, quinine, chloroquine, or hydroxychloroquine regulates protein acetylation events and the combination with all-trans retinoic acid stimulates histone acetyltransferase and inhibits HDAC enzymes in breast cancers (Figure 2). Nevertheless, the direct inhibition of the HDAC enzyme does not appear to be necessary for the differentiating activity of antimalarial quinolines [76].

Breast MCF-7 cells (wild-type for p53) presented 74% of cell cycle arrest in the G_1_ phase after 24 h and 72 h of exposure to chloroquine 50 *μ*M and everolimus [20 nM, 40-*O*-(2-hydroxyethyl)-rapamycin, an mTOR inhibitor], showing additive inhibitory effects when both drugs were added in 3-D cocultures. This proliferative reduction was confirmed by DNA quantification and increased levels of p53 and p21^Cip1^ after the treatment of MCF-7 cells with chloroquine, but not everolimus, which indicates that *G*_1_ arrest is mediated by tumor suppressor pathways p53 and p21 [48].

Loehberg et al. [46] detailed the dependency of p53 on the effects of chloroquine on BALB/c p53-null mammary epithelium cells and human mammary gland epithelial MCF10A line. Chloroquine-dependent DNA damage activates p53 and its downstream gene p21, resulting in the G_1_ cell cycle arrest after a post-translation p53 activation by chloroquine-induced phosphorylation of ATM proteins, proving the existence of ATM-dependent phosphorylation of the p53 checkpoint (Figure 2). These molecular findings may explain the particular ability of chloroquine 3.5 mg/kg to reduce the growth rate and tumor incidence by 41% only in p53-wild-type BALB/c mice exposed to *N*-methyl-*N*-nitrosourea after 8 weeks of treatment. Since the TP53 is a mediator of hormone (estrogen/progesterone)-induced protection against chemical mammary carcinogenesis and no protection was observed in BALB/c p53-null mammary epithelium, it certainly shows that chloroquine can prevent breast cancer similar to estrogen/progesterone treatment and shows a p53 dependence [46].

As described before, autophagy is required for efficient growth of cells, and upon starvation chloroquine decreases LC3II lysosomal degradation [66, 73, 78, 79]. Therefore, 67-NR and 4-T1 mouse breast cell lines treated with chloroquine were sensitized preferentially in response to phosphoinositide 3-kinases or mTOR inhibitors, the route that directly regulates autophagy (Figure 3). Surprisingly, chloroquine sensitized 4-T1 and 67-NR cells to inhibit phosphoinositide 3-kinases or rapamycin even in *Atg12* gene nonfunctional cells, and the pan-caspase inhibitor zVAD-fmk (zVAD) did not increase cell survival, indicating that chloroquine should be able to sensitize even when autophagy has already been previously obstructed. Corroborating these findings, decreasing the cell viability involves a caspase-independent mechanism in which chloroquine but not bafilomycin A1 sensitizes cells to rapamycin-mediated cytotoxic actions, even though both of them block autophagy and LC3-II degradation [53].

#### 2.2.5. Lung Cancers

Low concentrations of chloroquine (0.25–32 *μ*M) up to 24 h exposure induced apoptosis of adenocarcinoma lung A-549 cells and vacuolation with increased volume of acidic compartments, but caused necrosis at 48 h and higher concentrations, as demonstrated by lactate dehydrogenase assays. Interestingly, in the presence of D609, a specific inhibitor of phosphatidylcholine-specific phospholipase C, only lower concentration effects were suppressed [80].

Hu et al. [50], using screening cytotoxic methods and absorbance assays, pointed out that the coculture of chloroquine and Akt inhibitors (phosphatidylinositol analogs, oligopeptides Akt-PH linkers, direct inhibitors of Akt-kinase activity, and blockers of catalytic subunit in the ATP-binding site) are more effective than either one alone. Such killing effects of chloroquine-mediated chemosensitization occurs at low concentrations as 10–20 *μ*M and present specificity up to 120-fold for killing cancer than normal cells [50]. These findings indicate that chloroquine might significantly increase the therapeutic effects of some PI3K-Akt inhibitors with minor action on immortalized normal mammary gland epithelium 184B5 cells. Probably, chloroquine-mediated chemosensitization is related to the ability to block the formation of digestive vesicles, as those activated by tephrosin on cells, a natural rotenoid that induces endocytosis and subsequent degradation of human epithelial tyrosine kinase (HER-1 and 2) receptors [81].

#### 2.2.6. Melanomas

A screening chemical library of antimalarial drugs against melanomas showed the endoperoxide-based redox antimalarial artemisinin-class members as inducers of apoptosis, while metastatic melanoma cells (A375, G361, and LOX) displayed a specific vulnerability to artemisinin and semisynthetic artemisinin-derivatives and NOXA-dependent apoptosis [82], a proapoptotic member of the Bcl2 family. Such sensitivity was corroborated by the upregulation of cellular oxidative stress, phosphatidylserine externalization, and cleavage of procaspase-3 [82]. Next, amodiaquine-exposed A-375 and G361 melanoma cells exhibited the formation of multivesicular single membrane-enclosed structures with electron-dense inclusions (indicative of lysosomal expansion), impairment of mitochondrial transmembrane potential, and accumulation of LAMP1, p62/SQSTM1, *α*-synuclein, lipofuscin, and LC3-II at concentrations as low as 1 *μ*M [57], all accumulating autophagic proteins as a consequence of blocked autophagic-lysosomal flux (Figure 3). Such a blockade revealed a similar pattern of impaired lysosomal acidification in response to the treatment with either bafilomycin A1, amodiaquine, and chloroquine from a mechanistic point of view.

Intriguingly, a comparative analysis performed in A375 melanoma cells showed higher antiproliferative activity of amodiaquine when compared to chloroquine, which was confirmed by array analysis, revealing the modulation of gene expression antagonizing cell cycle progression (upregulation of *CDKN1A* and downregulation of *E2F1*) and modulation of the genes *TP53*, *CDKN1A*, *E2F1*, *CCND1*, and phosphorylated RB1. On the other hand, chloroquine failed to alter protein levels of TP53, E2F1, CCND1, and HSPA1A in A375 cells, demonstrating that the chloroquine treatment was not associated with the induction of cell cycle arrest, a finding extremely different from amodiaquine-induced melanoma cell cycle obstruction in the *S* phase [57].

Previous studies had already indicated amodiaquine as a more potent antimalarial molecule than chloroquine, a property attributed to a tropism targeting the acidic food vacuole of the plasmodium parasite [83]. Amodiaquine is a lysosomotropic 4-aminoquinoline-based tertiary amine as well, but it has a 1,4-aminophenol-pharmacophoric substituent capable of forming an electrophilic quinoneimine-metabolite under intracellular conditions of oxidation. Then, this reactive intermediate induces covalent protein adductions [57] and may contribute to higher potency.

#### 2.2.7. Retinal Pigment Epithelial Cells

10–250 *μ*M chloroquine produced a persistent reduction in mTOR activity and intracellular calcium in retinal ARPE-19 cells, leading to the nuclear translocation of transcriptional factors for lysosomal biogenesis, expansion of lysosomes, severe suppression of autophagosome-lysosome fusion, and increased cytosolic levels of LAMP1, beclin-1, glyceraldehyde 3-phosphate dehydrogenase (GAPDH), and phospholipid intracellular content in 25-fold or greater [40, 84].

The inhibitors of endocytosis reduce endosomes and arrest a considerable amount of GAPDH into lysosomal cytosolic vesicles and cell membranes. On the other hand, its degradation is physiologically reduced or blocked as an adaptive reaction of lysosomes to retrieve normal functions, although accumulation of intracellular substrates, including p62, GAPDH, and phospholipids, are not entirely reestablished [84]. Anyway, chloroquine-induced protein accumulation indicates autophagy inhibition because p62 and GAPDH are degraded by lysosomes via autophagy and chaperon-mediated autophagy pathways, respectively [85].

GAPDH, an enzymatic 144-kDa tetramer expressed on the cell surface and secreted from cells leading to forward trafficking of active GAPDH out of cells, actively contributes to endosomal recruitment [85]. The versatility and promiscuity of functions and its interaction with multiple protein partners make GAPDH a vital tool for cell survival because it works as a scavenger agent to flush out misfolded molecules and activates inside processes during membrane trafficking and production of secretory lysosomes [86].

#### 2.2.8. Mouse Embryonic Fibroblasts

In mouse embryonic fibroblasts (MEF), chloroquine and hydroxychloroquine confirmed their capacity to block autophagy in a concentration-dependent manner [87]. Indeed, Bax^−/−^ and Bak^−/−^ MEF cells were resistant against hydroxychloroquine-induced mitochondrial and plasma membrane permeabilization and hydroxychloroquine induced cathepsin B intracellular redistribution (Figure 3); besides, it was unable to cause mitochondrial depolarization, release of cytochrome *c*, or cell death when compared to wild-type MEF cells. Altogether, these data imply a specific sequence of subcellular alterations: (a) lysosomal accumulation resulting in the selective loss of mitochondrial potential and release of lysosomal enzymes, such as cathepsin B; (b) activation of Bax and mitochondrial permeabilization, and (c) caspase-3 activation, phosphatidylserine exposure, chromatin condensation, DNA loss, and apoptosis (Figure 2) [36]. Correspondingly, *in vivo* effects following 24 h or 48 h exposure of C57BL/6JOlaHsd mice to hydroxychloroquine 60 mg/kg showed Golgi changes and accumulation of LC3 [87].

These cells were also tested with a panel of approved-FDA drugs containing either quinoline or quinolone pharmacophores. Chloroquine caused the secretion of prostate apoptosis response-4 (Par-4) from wild-type p53 MEFs (Figure 2), as well as from normal human prostate stromal and lung fibroblast cells and their respective aminoquinoline derivatives, and induced Par-4 systemic secretion in C57BL/6 mice in a dose of 50 mg/kg body weight, and in patients from a clinical trial against cancer prior to surgery taking hydroxychloroquine [88]. As predictable, chloroquine caused the accumulation of LC-3II and p62/SQSTM1, but drug-induced secretion of Par-4 was not inhibited by zVAD, and differences in p62 levels have not been noticed after the treatment with wild-type Par-4 and Par-4^−/−^ cells [88].

Par-4 is a tumor suppressor capable of inducing apoptosis selectively in most cancer cells without affecting normal/immortalized/nontransformed ones. The increase of Par-4 in the extracellular matrix causes cell death of tumor cells through binding to the overexpressing GRP78 receptor on the cell surface. Normal lines exhibit undetectable-to-low levels of this receptor [89], which protect them from the “friendly fire” though Par-4 is secreted by both normal and cancer tissues [89, 90]. Therefore, Par-4 secretion is not associated with apoptosis and does not affect autophagy in normal mouse embryonic fibroblasts. Meanwhile, the cocultures of chloroquine-treated Par-4^+/+^ MEFs plus H-460 lung p53^+/+^ and H-1299, HOP92, and KP-7B lung or prostate PC-3 p53^−/−^ cancer cells were sensitive to apoptosis, but not when cocultured with chloroquine-treated Par-4^−/−^ MEFs, and chloroquine failed to induce Par-4 secretion in prostate cancer cells (LNCaP, C42B, DU-145, and PC-3) and lung cancer cells (H-460 and A-549). These discoveries indicate that chloroquine-induced Par-4 secretion from normal lines causes paracrine apoptosis in cancer cells [86], and such action increases the selective expression of Par-4 receptor GRP78 on the surface of cancer cells [91] (Figure 2).

*In vivo* related findings in C57BL/6 mice bearing LLC1 pulmonary tumors also showed systemic elevation of Par-4 regressed tumor growth and metastatic lung nodules in animals treated with chloroquine 25 mg/kg/days for 5 consecutive days [88, 90]. Once again, the antiproliferative activity of chloroquine is linked to the activation of p53 and inhibition of NF-*κ*B because these events promote Par-4 secretion [88] because p53 regulates classical components of the secretory route (Figure 2). This relatively unknown Par-4 pathway adds new importance to the traditional DNA protection roles of normal *TP53* gene to manage the tumor suppressor.

### 2.3. We Need to Think Outside the Box

#### 2.3.1. New Pharmacological Judgement

Traditional comprehension about the effects of chloroquine and analogues on the lysosomal physiology implies a specific sequence of subcellular alterations: (a) lysosomal accumulation resulting in the selective release of lysosomal enzymes, such as cathepsin B and D; (b) activation of Bax/Bad and mitochondrial permeabilization; (c) loss of mitochondrial potential, and (d) activation of caspases, phosphatidylserine exposure, chromatin condensation, DNA loss, and apoptosis (Figure 2).

However, new pharmacological judgements have arisen and changed some scientific dogmas in this area. Higher lysosomal pH was observed after 4 h of treatment with known alkalinizer drugs (fluoxetine, imipramine, dimebon, tamoxifen, chlorpromazine, amitriptyline, and verapamil), including chloroquine. Considering their high lipophilic structures (clogP ranging from 3.49 to 6.24), this suggests suitable entry into target cells including osteosarcoma U2OS, adenocarcinoma cervical HeLa, embryonic rat cardiomyocytes H9C2, and the human retinal pigment epithelial ARPE-19 line. Indeed, among amodiaquine, artemisinin, mefloquine, piperaquine, primaquine, quinacrine, and chloroquine, two antimalarial compounds (mefloquine and quinacrine) were about 30- and 60-fold more potent autophagy inhibitors on U2OS cells than chloroquine, respectively [92].

However, higher pH values were sustained no more than the compound exposure time, and after 24 h, renewed acidic organelles with pH between 4-5 were detected, indicating restorage of pH, which was also confirmed by nuclear translocation of transcription factors involved in lysosomal biogenesis, bigger lysosomal volume, and returning of cathepsin levels in order to reestablish optimal conditions for enzyme digestion [84, 93, 94].

Most studies have also suggested that chloroquine- or hydroxychloroquine-induced cell death is initiated by the “type II programmed autophagic/lysosomal pathway,” including sequestration of organelles into autophagosomes and cytoplasmic vacuolization (Figure 3), and these processes are followed by later signs of the “type I programmed death” [36, 40, 66], which traditionally display karyorrhexis, DNA fragmentation, release of mitochondrial cytochrome *c*, activation of Bcl-2 proapoptotic proteins and caspases, cellular shrinkage, and phosphatidylserine externalization (Figure 2). Additionally, although members of the 4-aminoquinoline family, including chloroquine, hydroxychloroquine, and Lys-05 (dimeric chloroquine) inhibit autophagy [68], it has been suggested that ribosome biogenesis stress found in treated cells is not a general consequence of autophagy inhibition and that amodiaquine stands out among the 4-aminoquinoline family as a compound functioning by 2 independent mechanisms in two distinct intracellular environments: cytoplasm, where autophagy inhibition occurs and nucleolus, for diminution/blockage of ribosome biogenesis [49], which demonstrate that amodiaquine but not chloroquine inhibits ribosome biogenesis, disrupts nucleolar structure, and triggers degradation of RNA polymerase I.

As endosomal trafficking, endosome-lysosome fusion, membrane stability, signaling pathways, and transcriptional activity are impaired by hydroxychloroquine and chloroquine, it was hypothesized that combining them with radiation would be a good adjuvant alternative [47]. Nevertheless, chloroquine sensitization of some breast cancer lines revealed to be independent of autophagy inhibition, since sensitization was not mimicked by the knockdown of *Atg12* or *Beclin 1* genes or following treatment with bafilomycin A1, and chloroquine-induced cell death occurred even in the absence of Atg12 [53], proposing that reducing autophagy does not affect drug cytotoxicity ubiquitously in all human cells. Meanwhile, studies have demonstrated that chloroquine has specific cell sensitization effects to particular antimitotic drugs, whereas primaquine and mefloquine can sensitize resistant cancer cells to all antimitotic drugs without preference [81].

In a similar way, most investigations indicate that chloroquine does not block all forms and steps of the endolysosomal system. Analysis showed that chloroquine/hydroxychloroquine inhibits autophagy in initial phases, causing accumulation of acidic vesicular organelles and break/discontinue autophagosome-lysosome fusions, but they do not alter the ability of lysosomes to digest target macromolecules as conventionally accepted. In another point of view, compounds that simply increase the upstream autophagic flux without altering downstream fusion and degradation steps may not provide therapeutic benefit [95]. This would explain why only chloroquine and hydroxychloroquine are officially recommended as autophagy inhibitors by the Food and Drug Administration (FDA).

If we recall a more integrated concept, considering the well-established details about the blockage of autophagic flux and the capacity to inhibit PI3K/Akt/mTOR pathways and trigger ATM/ATR/p53/p21 signaling, it is possible to visualize that they complement themselves to cause death of cancer cells. Once mTOR is commonly phosphorylated at position 2448 via the PI3K/Akt and has been inhibited when higher levels of p15^INK4B^, p16 ^INK4A^, p21^Cip1^, p27^Kip1^, p53, and other suppressor tumors are present under stress conditions, p21 obliges *G*_1_ restriction by inhibitory binding to CDK2/cyclin E or other CDK/cyclin complexes [45]. These physiological aspects support the theory that chloroquine or hydroxychloroquine exhibits, at least in part, antineoplastic effects altering the phosphorylation status of EGFR/PI3K/Akt/mTOR/Atg and p53 pathways [43, 46, 48, 49, 57] due to the direct inhibition of PI3K-Akt kinases, obstruction of catalytic subunits in the ATP-binding site [80], and/or misregulation of signaling of epithelial growth factor receptors (EGFRs) during endocytosis because they seem to weaken receptor-mediated endocytic transfers of TKRs to degradative compartments [95] (Figure 3).

In this context, the inhibition of tyrosine kinase receptors and downstream pathways (Receptor/PI3K/Akt or Receptor/Grb2/Ras/Raf/MEK/ERK) are examples of suitable targets to select antitumor repurposing molecules [50, 80, 81] (Figure 3). Despite that tyrosine kinase inhibitors have demonstrated enhanced selectivity, extra effects on some kinases and beyond their target family show intrinsic polypharmacology often favorable for clinical efficacy [6]. Therefore, blocking the signaling pathways that maintain the stemness is thus a rational goal to avoid recurrence as well as to block tumor growth and metastasis. Metastatic cancer or surgically nonresectable tumors show five years mortality above 90% in aggressive cancers, e.g., pancreatic tumors and acute myeloid leukemia. Hence, with a few exceptions, survival rates of aggressive cancer types are low, mainly due to therapeutic failure [15].

Instinctively, these new studies indicate that chloroquine does not increase lysosomal alkalinization in all cell types in a similar magnitude, and lysosomes may even maintain their competence (completely or not) to digest organic material, confirming that chloroquine inhibits the fusion between autophagosomes and lysosomes in a concentration-dependent way, but it does not change the lysosomal activity considerably [84, 87]. The extent of increase in lysosomal pH and how much time lysosomes demand to normalize after compound exposure can diverge a lot if we take into consideration cell specificities, doubling time, phagocyte capacity, and how efficiently the cells/lysosomes respond to the compound sequestration. In a cell point of view, autophagy responses constitute stress adaptation that can suppress apoptosis, but when autophagy is blocked either at earlier or later stages, it may lead to apoptosis as a result of the failure for adaptation to environmental changed states.

Overall, the precise mechanism by which quinines sensitizes cancer cells by PI3K-Akt or MEK/ERK inhibitors is unclear, but it is recognized that such signal pathways are overexpressed or upregulated in cancer rather than in normal cells, which opens a “window of opportunities” to design more target drugs and clinical trials based on the lysosomal blockade ability. It is very important to remember that patients with metastases present tumors with multiple molecular and cellular characteristics. Therefore, the heterogeneity of metastases, tumor advance, and cell selection becomes a common problem observed in tumor resistance during the first line chemotherapies [6]. Therefore, including sensitizers with antimutagenic action (such as chloroquine) reduces the extent of primary DNA rearrangements responsible for the appearance of mutant clones and may delay/inhibit tumor progression.

A generalized overview also emphasizes the most vulnerable issue: do the effects of aminoquinolines share a common mode of action or are they the products of a variety of distinct processes? Once their mechanisms remain uncertain, molecular and clinical lessons are indispensable to detail dose/concentration-response relationships and safety-related aspects to guide the development of new modulating autophagy therapies [30].

#### 2.3.2. Pharmacokinetic-Related Toxicity

The most common adverse effects of chloroquine, hydroxychloroquine, amodiaquine, and other aminoquinolines in clinical use are nausea, abdominal/hypochondrial pain, changes in visual acuity (blurred vision), bitter taste in mouth, insomnia, weakness, arthralgia, back pain, pruritus (sensation of itching and stinging), diarrhea, and pale stools. Indeed, up to 50% of patients receiving hydroxychloroquine report some gastrointestinal effects. This is dose-dependent and most often occurs with loading doses >800 mg [96, 97], but 400–800 mg daily doses have been related to symptoms of psychosis, agitation, insomnia, confusion, hallucinations, paranoia, depression, catatonia, and suicides. These psychological/neurological effects may appear at any age, during acute or chronic use, and in patients with or without a history of psychiatric illness [98].

Poisoning with antimalarial drugs have also caused cardiovascular problems such as myocarditis, ventricular arrhythmias, cardiac arrest, and QTc prolongation due to the blockade of hERG potassium channels. Thereof, chloroquine, hydroxychloroquine, amodiaquine, and other derivatives should be used with caution in oncologic patients with cardiac diseases, history of ventricular arrhythmias, hypokalemia and/or hypomagnesemia, or bradycardia (˂50 bpm), and during concomitant administration of QT interval prolonging agents (e.g., macrolides and fluoroquinolones) [9, 99–101]. If cardiotoxicity is suspected, quick discontinuation of the QT interval prolonging agents may prevent life-threatening complications.

Severe hypoglycemia with the loss of consciousness in patients treated or not with antidiabetic medications have been observed [102], inspite of beneficial effects for the metabolic syndrome [103]. Therefore, patients presenting clinical symptoms of hypoglycemia during treatment should have their blood glucose checked and treatment reviewed when necessary. Additionally, rhabdomyolysis [99] and ototoxicity when they are used by pregnant women in the 3rd trimester and even irreversible deafness [104] were reported. Nonetheless, health guidelines have indicated the maintenance of treatment with hydroxychloroquine during pregnancy and breastfeeding in patients with autoimmune diseases since these aminoquinolines do not cross the placenta easily and low quantity is found in breast milk [105].

Chloroquine and hydroxychloroquine structures and modes of action are closely similar except for an additional hydroxyl moiety, which makes hydroxychloroquine less permeable to blood-retinal barrier, and it allows faster clearance from retinal pigment cell, suggesting minor risks and safer option since long-term clinical trials with hydroxychloroquine tolerates higher daily doses and revealed less drug-drug interactions [102]. Their therapeutic window is relatively narrow, and retinal damage is one of the most common side effects for long term use [106]. Around 20% of chloroquine users showed ocular injuries due to high doses and treatment frequency in 1980s [39], and, since 1974, it has been a prescribed medicine in Japan due to chloroquine-associated retinopathy [107]. Thus, ocular or color vision examinations of patients under antimalarial therapies is indispensable for the early detection of retinal toxicity at a stage in which it is still reversible once treatment is interrupted [39]. The initial development of retinal damages with a daily dose of 800 to 1200 mg of hydroxychloroquine has been detected using sensitive retinal screening tests [108].

**Figure 4:**
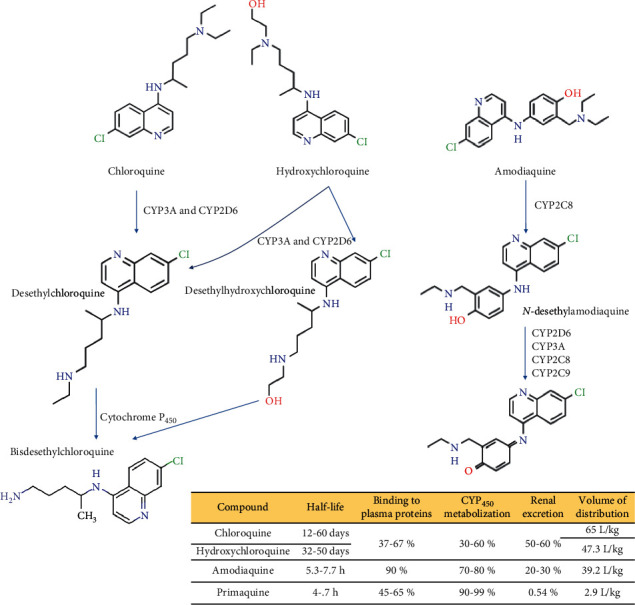
Metabolism of some aminoquinolines and pharmacokinetic features.

The simultaneous use of tamoxifen—the most prescribed selective modulator of estrogen receptors to treat hormone-receptor-positive, early/advanced-stage or metastatic breast cancers after surgery to reduce the risk of recurring—with hydroxychloroquine increases the risk of eye toxicity owing to the synergistic block of lysosomal/autophagy steps in retinal epithelial cells and accumulation of potentially toxic ubiquitinated proteins [108]. Although retinopathy is more commonly correlated with chloroquine than with hydroxychloroquine, which might also be explained by the lower volume of distribution (Vd) for hydroxychloroquine (47.3 L) compared with chloroquine (65 L) (Figure 4). Ophthalmology guidelines have recommended comedication of tamoxifen plus hydroxychloroquine for up to 6 months, and a maximal daily dose of 5 mg/kg/day body weight of hydroxychloroquine not more than 5 years [30, 40, 106, 109].

Besides molecular similarities, chloroquine and hydroxychloroquine occur as enantiomers (R and S isomers), and *in vitro* and *in vivo* analyses have not shown important differences owing to the bioactivity [30], stereoselectivity of drug-drug interactions, and clinical consequences on toxicity due to the preferential metabolism of one enantiomer [105]. Both S(+)-chloroquine and -hydroxychloroquine present higher binding to albumin and *α*_1_-acid glycoprotein, but hydroxychloroquine was enantioselective *in vivo* and in healthy volunteers, indicating the less protein-bound R(−)-enantiomer [110]. Then, the hydroxychloroquine binding degree to plasma proteins seems to control its distribution into cells, which can help explain how chloroquine have a larger Vd, since its R(−)-enantiomer is almost 2-fold less protein bound than the S(+)-enantiomer [105]. Notably, the S(+)-form of hydroxychloroquine is less taken up by rabbit ocular tissues [111], which suggests that the administration of the pure S(+)-enantiomer could offer better efficacy and lesser toxicity [105].

Experimental blockers such as 3-MA, bafilomycin A1, and short hairpin RNA (shRNA) knockdown of gene *Beclin* cause the deficiency of autophagy and increase tubular cell p53-dependent apoptosis during cisplatin treatment in kidney proximal tubular cells [112], supporting convincing data that autophagy is critical for renal cell survival. Hence, the concomitant exposure to anticancer agents and clinically available autophagy blockers (e.g., amodiaquine, primaquine, and analogues) also sensitizes normal tissues and can dramatically worsen renal function in patients with acute or chronic kidney illnesses. Under these circumstances, an impaired renal function increases the bioavailability of antimalarial drugs and comedications and the risk of adverse effects by pharmacological interactions.

In the pharmacokinetic context: (i) a single oral dose of chloroquine 300 mg can be detected in blood and urine from healthy volunteers up to 52 and 119 days postdose, respectively [113]; (ii) terminal elimination half-life of chloroquine, hydroxychloroquine, and their active metabolites (desethylchloroquine and desethylhydroxychloroquine, respectively, and finally, bisdesethyl chloroquine as a downstream metabolite of both drugs) varies from 20–60 days [30, 114]; (iii) both drugs can distribute to aqueous cellular and intercellular compartments, resulting in long mean residence times (about 1,300 h for hydroxychloroquine and 900 h for chloroquine) [114]; (iv) 30 to 50% of these antimalarial drugs are transformed by hepatic cytochromes P_450_, mainly, CYP3A and CYP2D6 [115], and (v) about 37–67% of chloroquine/hydroxychloroquine bound to liver-derived plasma proteins [110, 116, 117]. Additionally, the half-life of amodiaquine is only 5.3–7.7 h, since it is subject to rapid first-pass metabolism and generate *N*-desethylamodiaquine, the principal route of disposition in humans, whose active metabolite has half-life >100 h and, therefore, amodiaquine can be considered a prodrug [118]. Thus, in contrast to amodiaquine, chloroquine and hydroxychloroquine are not highly bound to plasma proteins but have strong tissue binding.

It is also critical to ponder the coexistence of hepatic diseases (cancer-related or not), first pass metabolism, and comedications if the question is bioavailability or linked-side effects because elimination is significantly reduced in the presence of hepatic dysfunction, and nearly 50% of chloroquine is recovered in urine as unchanged drug. As background, a recent Brazilian study showed that the administration of hydroxychloroquine (400 mg twice daily for 7 days) without or with azithromycin (500 mg once a day for 7 days) caused a rise in liver-enzyme levels [119].

Indeed, clinical trials with anticancer purposes showed that adverse effects and toxicity of chloroquine and hydroxychloroquine are strongly dose-dependent (100–1200 mg/day). According to Common Terminology Criteria for Adverse Events version 3.0, toxicity was found with 100 to 200 mg/day [120–122]. Between 200 and 600 mg/day, the most common adverse effects were classified as grade 1 and 2, and include rash, visual blurring, sensitivity to light, nausea, diarrhea, fatigue, weight loss, vomiting, dyspepsia, anorexia, and dry skin [123–128]. The adverse effects of grade 3 or higher were detected from 600 to 1200 mg/day. Grade 4 toxicity was associated with myelosuppression at 800 mg/day of hydroxychloroquine [122]. Meanwhile, the combination of temsirolimus (25 mg/day, mTOR inhibitor) and hydroxychloroquine (200–1200 mg/day) was considered safe and tolerable, even at highest doses in patients with advanced solid tumors and melanoma [126]. Grade 2 or 3 adverse events were more common, resulting in a decrease of dosages after 2–3 months of treatment. Hydroxychloroquine and bortezomib, a proteasome inhibitor administered in patients with relapsed/refractory myeloma, cause grade 1 or 2 adverse events, mainly, but some patients experienced bone marrow suppression and grade 3 gastrointestinal toxicity [127]. At 1200 mg/day, hydroxychloroquine induced lymphopenia and an increase in serum alanine aminotransferase (grade 3/4) in patients with metastatic pancreatic cancer [128].

## 3. Conclusions

The mechanisms of sensitization attributed to aminoquinolines have a histological basis, but most of them are interconnected to the autophagic process. They express signals of autophagy disruption and cytotoxic-related action, including accumulation of key markers, predominantly, LAMP1, p62/SQSTM1, LC3 members, GAPDH, beclin-1/Atg6, *α*-synuclein, and granules of lipofuscin.

**Figure 5:**
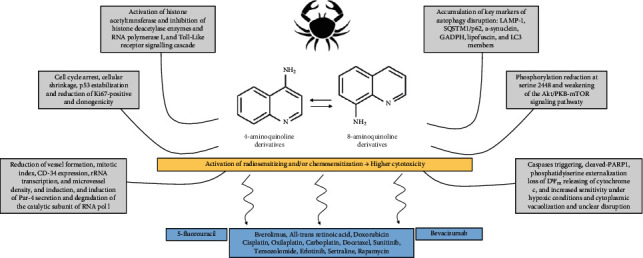
General properties of aminoquinolines on tumors.

Aminoquinolines act as lysosomal alkalinizers and take ownership during death-promoting mechanisms, which explain, at least in part, their chemotherapy and radiotherapy sensitizer effects when used as adjuvant option in clinical trials against solid tumors. They overturn lysosomal-related pathophysiological barriers, reduces uptake and drug distribution, avoid resistance, and improve cytotoxic activity response of weak-base clinical drugs, since they work as chemosensitizers under specific microenvironmental conditions, especially when acid lysosomal and inflamed tissues pH cause ion trapping and sequestering of chemotherapeutic drugs into protonated acidic endosomes. Additionally, they have also overwhelmed tumor resistance *in vivo*, suggesting that autophagy inhibition has antiangiogenic effectiveness as well. Therefore, in a mechanistic point of view, aminoquinolines induce ATM-ATR/p53/p21 signaling, caspase activation, and exhibit unspecific capacity for overlapping the apoptotic cascade to either upstream of caspase-3 activation and/or encompass nonp53/apoptotic/autophagy routes (Figure 5).

More specifically, two 4-aminoquinolines—chloroquine and hydroxychloroquine—accumulate slowly into cells and take time to develop cytotoxicity. Then, longer time exposure is believed to provide better antiproliferative effects, considering that they have a late onset but a prolonged action even after drug discontinuation. Moreover, no important differences have been found about the stereoselectivity of drug-drug interactions, clinical consequences on bioactivities, and additional pharmacokinetic-related toxicities. However, a continuous pharmacovigilance is required because these antimalarial molecules exhibit multiple cellular unspecific modes of action (undesired off-targets), relatively narrow therapeutic windows, recurrent adverse effects, and self-treatment-related poisoning. Retinopathy, mainly, has been more associated with chloroquine, and compromised renal and liver functions and increased the bioavailability of antimalarials and risk of adverse interactions. Therefore, their use must be under rigorous rules, ethical and medical prescription, and clinical and laboratory follow-ups.

## Figures and Tables

**Table 1:** General mechanisms of chemosensitizing and radiosensitizing adjuvant actions of chloroquine, hydroxychloroquine, and analogues.

Treatment/Drug	Adjuvant actions	References
Phosphatidylinositol analogs, oligopeptides Akt-PH linkers, inhibitors of Akt-kinase, and blockers of ATP-binding site catalytic subunit	Mediated chemosensitization and enhanced cytotoxicity	[50]
All-trans retinoic acid	Reduction of Ki67-positive cells and clonogenicity, activation of histone acetyltransferase, and inhibition of histone deacetylase enzymes	[51]
5-Fluorouracil	Down-regulation of CDK-2 expression and cyclin E/CDK2 complex activity, arrest in G_0_/G_1_ phase, and enhancement of antiproliferative properties	[52]
Everolimus	Proliferative reduction, increase of p53 and p21^Cip1^ levels, phosphorylation reduction at serine 2448 in mTOR proteins	[48]
Rapamycin	Blockade of autophagy and LC3-II degradation, cytotoxic chemosensitization and involvement of a caspase-independent mechanism	[53]
Cisplatin	Increase of caspase-3 activation, LC3 II ubiquitinated intracellular misfolded proteins, and intrinsic apoptosis	[54, 55]
Docetaxel	Enhanced cytotoxicity and stronger *in vivo* anti-tumor efficacy	[56]
Doxorubicin	Potentiated cytotoxicity upon coexposure	[57]
Oxilaplatin	Increased sensitivity under hypoxic conditions and p62 levels, delaying of tumor growth of HT-29 colon cancer xenografts	[58]
Sunitinib	Increase of the p62 level, reduction of blood vessel formation, CD-34 expression, microvessel density, and nitric oxide levels in tumor, and Ehrlich ascites carcinoma tumor growth reduction	[59]
Temozolomide	Cell viability reduction and intensification of cleaved-caspase-3, cleaved-PARP1, phosphatidylserine externalization, and caspase-3/7 activation	[54, 60]
Receptor-interacting protein kinase 3 (RIP3)	Upregulation of RIP3, accumulation of RIP3-p62 complexes and type II-LC3B, and efficiency on colon tumor-bearing mice	[61]
Bevacizumab	Weakening of the Akt-mTOR signaling pathway and recovering the tumor-suppressive effect of bevacizumab	[62]
Sertraline + erlotinib	Amplification of caspase-independent autophagic cell death and mouse survival in orthotopic non-small cell lung cancer mouse models	[63]

## Data Availability

The data used to support the findings of this study are cited as references.

## Abbreviations

ATM-ATR: Ataxia telangiectasia-mutated/ataxia telangiectasia and Rad3-related kinases
Atg: Autophagy-related protein
CDK: Cyclin-dependent kinase
EGFR: Epithelial growth factor receptor
GAPDH: Glyceraldehyde 3-phosphate dehydrogenase
GRP78: Glucose regulated protein 78
LAMP1: Lysosomal associated membrane protein 1
MAPK: Mitogen-activated protein kinases, originally called ERK, extracellular signal-regulated kinases
LC3: Microtubule-associated protein 1A/1B-light chain 3
NF-*κ*B: Factor nuclear kappa B
Par-4: Prostate apoptosis response-4
p53: Protein 53
p62: Protein 62
PARP1: Poly-(ADP-ribose)-polymerase 1
PI3K: Phosphatidylinositol 3-kinases
SQSTM1: Sequestosome 1
S6K1: p70S6 kinase1
TLR: Toll-like receptor
TKR: Tyrosine kinase receptor
UACA: Uveal autoantigen with coiled-coil domains and ankyrin repeats
Vps34: Vesicular protein sorting 34
4E-BP: 4E-binding protein
5-FU: 5-Fluorouracil
zVAD-fmk: Benzyloxycarbonyl-Val-Ala-Asp-fluoromethyl ketone.
